# Dynamic and static angry faces influence time perception differently—Evidence from ERPs

**DOI:** 10.3389/fnins.2023.1124929

**Published:** 2023-01-19

**Authors:** Fangbing Qu, Xiaojia Shi, Jia Dai, Tianwen Gao, Hongyan Wang, Changwei Gu

**Affiliations:** ^1^College of Preschool Education, Capital Normal University, Beijing, China; ^2^Beijing No.4 Kindergarten, Beijing, China; ^3^Yangzhen Central Kindergarten, Beijing, China

**Keywords:** time perception, dynamic characteristic, angry face, event-related potentials, dynamic facial expression

## Abstract

The dynamic characteristics of facial expressions might affect time perception. Compared with static emotional faces, dynamic emotional faces are more intense, have higher ecological validity, and contain time series information, which may lead to time overestimation. In the present study, we aimed at investigating how dynamic characteristics of angry facial expressions affect time perception, as measured using event-related potentials (ERPs). Dynamic and static angry and neutral faces with different durations (400, 600, 800, 1000, 1200, 1400, and 1600 ms) were presented in the classical temporal bisection paradigm. Participants were asked to judge whether the duration of the presented face was closer to 400 or 1600 ms. The behavioral results showed a significant overestimation effect for dynamic angry faces compared with static faces, both in terms of proportion of long and Bisection Point. The ERP results indicated that the processing mechanisms are significantly different between judging the duration of dynamic and static angry faces. Dynamic angry faces evoked a larger N2 and Late Positive Potential than did static faces, while the static angry faces evoked a larger P2 and Early Posterior Negativity. The Contingent Negative Variation showed a complex change pattern over time. Our results indicate that dynamic angry facial expressions influence time perception differently than do static faces. Static angry faces were processed earlier and were considered to cause an overestimation of time through early emotional arousal and attentional bias, while dynamic angry faces may have caused the overestimation of time through response inhibition and late sustained attention.

## 1. Introduction

Time is the basic dimension of life. Rapid and accurate time perception greatly influences one’s daily life, especially in the context of social interaction. Failure to accurately perceive others’ facial expressions and responses that are too late or too early may result in social failure. According to previous studies, time perception can be greatly influenced by the emotional state of the social counterpart (Buhusi and Meck, 2005; Droit-Volet and Gil, 2009; Tamm et al., 2014). Perceived emotion may bias an individual’s time perception. In general, negative emotional events are perceived as longer than neutral or positive emotional events (known as the subjective lengthening effect), while happy emotions make people feel like “time is flying” (known as the subjective shortening effect) (Gil and Droit-Volet, 2011; Droit-Volet et al., 2016; Tian et al., 2018). Moreover, distinct negative emotions may exert different effects on time perception. Gil and Droit-Volet (2011) reported that anger, fear, and sadness have a lengthening effect, while shame has a shortening effect or no effect on time perception, depending on whether participants correctly recognized this emotion. The level of arousal induced by facial expression can also affect time perception; for instance, angry expressions have been found to result in a larger lengthening effect than sad expressions (Fayolle and Droit-Volet, 2014).

Another factor that can influence time perception is the dynamic (movement) feature of a stimulus. Static photographs and pictures are the most frequently used stimuli in previous studies (Gil and Droit-Volet, 2011; Li and Yuen, 2015). However, compared with static facial materials, dynamic faces are more natural, more common in daily life, and better reflect individuals’ genuine emotional states (Sato and Yoshikawa, 2007; Li and Yuen, 2015). Several studies have also shown that subjects judge the emotion of moving faces as more intense and realistic than that of static faces, and recognition accuracy is also reportedly enhanced for dynamic stimuli (Wehrle et al., 2000; Biele and Grakowska, 2006; Sato and Yoshikawa, 2007). Therefore, dynamic emotional stimuli may have a different effect on time perception than do static stimuli. According to the scalar timing theory, dynamic faces attract more attention than static faces, and thus lead to a more robust lengthening or overestimation effect (Gibbon, 1977; Gibbon et al., 1984). More recently, empirical research from Fayolle and Droit-Volet (2014) examined the effects of dynamic facial expression displays on time perception in a temporal bisection task. The participants were firstly trained to respond “short” or “long” after presented the short (0.4 s) and the long (1.6 s) standard duration in the form of an oval. They were then presented with seven different comparison durations and asked to respond whether the comparison duration was more similar to the “short” or “long” standard duration. In the formal test, the oval was replaced with different arousing emotional facial expressions (anger vs. sadness) in either a dynamic or a static form with different comparison durations. Their results suggested that facial movements amplified the effect of emotion on time perception, whereby dynamic angry emotional expressions were perceived as being longer than static sad expressions.

The specific effect of facial dynamic features on time perception might have different neural underpinnings to the effect of static features. Previous work on the neural mechanisms underlying facial emotion processing has shown that dynamic emotional face processing is mainly associated with activity in brain areas related to social treatment (the superior temporal sulcus) and to emotion processing (the amygdala) (Alves, 2013). Previous studies have also tested the electrophysiological indicators of time perception during the presentation of static faces (Dan et al., 2009; Gan et al., 2009; Tamm et al., 2014; Recio et al., 2017). However, to the best of our knowledge, no studies have yet investigated the neural correlates of the potentially distinct mechanisms underlying the effect of dynamic and static stimuli on time perception. Our study is therefore the first to examine this question using an event-related potential (ERP) methodology.

In accordance with previous studies on the neural mechanisms underlying the effect of emotion on time perception (Dan et al., 2009; Gan et al., 2009; Kei et al., 2011; Tamm et al., 2014; Recio et al., 2017; Wang et al., 2019), we focused our analyses on four ERP components, as follows: the P2, Early Posterior Negativity (EPN), Late Positive Potential (LPP), and Contingent Negative Variation (CNV). As an important early visual component, the P2 (100–200 ms) reflects a person’s sensitivity to emotional expression, and is affected by the interaction between emotional stimulation and task-related factors (Foti et al., 2009). In addition to these early visual components, the EPN and LPP are also often found and discussed in the context of emotional expression processing tasks. The EPN component reflects attentional processing of emotional information, but also is associated with the rapid detection of facial information. Previous studies have shown that threatening angry faces induce a larger EPN component in the early stage of expression recognition (Recio et al., 2011, 2017). The late LPP component has been shown to be associated with arousal estimation of upcoming emotional stimuli. LPPs induced by high-arousal faces are significantly greater in amplitude than those elicited by low-arousal faces, which indicates that high-arousal faces attract more attention and receive more processing resources. Compared with static faces, dynamic faces attract more attention, and once the dynamic characteristics are noticed, it is difficult to get rid of it, so this results in greater LPP volatility (Zhu and Liu, 2014). The CNV is considered to be a marker of time accumulation, target duration, and electrophysiological correlates of the perceived target duration. Some studies have demonstrated there to be a positive correlation between the average CNV amplitude and the estimated stimulus duration (Macar and Vidal, 2004; Gan et al., 2009; Tarantino et al., 2010; Kei et al., 2011). However, the evidence for this is inconsistent, with some work reporting there to be no direct relationship between them (Kononowicz and Hedderik, 2011; Tamm et al., 2014). This question needs to be further explored. Furthermore, the CNV amplitude has been found to be significantly correlated with different attentional resources recruited by different emotions. Compared with neutral faces, the CNV amplitude is smaller in response to faces exhibiting happiness and anger, because emotional processing lessens the cognitive resources allocated to time perception (Gan et al., 2009; Zhang et al., 2014).

Based on the above evidence, we investigated the temporal mechanisms underlying the effect of dynamic features of facial expression on time perception by employing the temporal bisection paradigm. Facial stimuli (angry vs. neutral) with different dynamic characteristic (dynamic vs. static) and durations (400, 600, 800, 1000, 1200, 1400, and 1600 ms) were presented and participants’ behavior performance and event-related potential (ERP) responses were recorded. We expected that the dynamic facial expression would be judged longer than static facial expression in behavior result, as previously evidenced as overestimation effect. Besides, the effect of facial dynamic features on time perception would also reflect on different EPR components evoked when processing dynamic angry faces compared with static angry or neutral faces. Based on previous literatures of the psychological meaning associated with different ERP components, we speculate that the amplitude of the timing sensitive CNV would display separated waveforms in different duration and dynamic facial expression conditions. Furthermore, compared with neutral and angry static faces, we predicted that dynamic angry faces would evoke larger amplitudes of the P2, EPN, LPP, and CNV components, indicating the presence of distinct neural mechanisms underlying the attention effects of different dynamic features of emotional expressions on time perception.

## 2. Materials and methods

### 2.1. Participants

Participants were 15 college students from a university in Beijing (*M* = 22.6 years old, *SD* = 1.61 years, 11 female). Participants were right-handed, had normal or corrected-to-normal vision, were not colorblind, and had no history of mental illness. The Research Ethics Committee of College of Preschool Education, Capital Normal University approved this study. Participants signed an informed consent form before the experiment, and were given an appropriate remuneration after the experiment.

### 2.2. Stimulus materials

We selected 20 photographs of neutral and static angry face from 20 models from the Nimstim database (Tottenham et al., 2009), and asked 20 college students (10 men, 10 women) to rate each for their emotion type and arousal on a Likert scale ranging from 1 to 9. Finally, the neutral and corresponding angry photos of 6 models were selected. The dynamic anger face was generated using fantamorph software, as in previous research (Fayolle and Droit-Volet, 2014). Each dynamic face was morphed from the neutral to the angry face of the same model. Each dynamic facial sequence consisted of 6 frames, with the duration of each frame depending on the total duration of the dynamic face (400, 600, 800, 1000, 1200, 1400, and 1600 ms) based on previous studies (Droit-Volet and Wearden, 2002; Gil and Droit-Volet, 2012; Fayolle and Droit-Volet, 2014). For instance, a dynamic facial sequence of 400 ms would consist six frames (each frame last for 66.6 ms), changing from a neutral face (the first frame) gradually to the last and most intense angry face frame (the sixth frame). Another 20 college students (10 men, 10 women) rated the emotion type and arousal of all three groups of stimuli (static neutral faces, static angry faces, and dynamic angry faces). The dynamic angry face stimuli were had one of three durations (400, 1000, and 1600 ms).

Recognition and arousal ratings for the three facial stimuli groups are shown in Table 1. There was no significant difference in recognition rate between the three groups, *F*(2, 357) = 0.08, *p* > 0.05. However, there was a significant between-group difference in arousal ratings, *F*(2, 357) = 155.14, *p* < 0.001, whereby the static and dynamic angry faces were rated as more arousing than neutral faces. No significant difference in arousal was found between static and dynamic angry faces.

**TABLE 1:** Description of ratings of different facial stimuli (*M* ± *SD*).

	Neutral face (NF)	Static angry face (SA)	Dynamic angry face (DA)
Recognition rate	0.84 ± 0.37	0.83 ± 0.38	0.84 ± 0.37
Arousal	3.33 ± 2.14	6.80 ± 1.93	6.97 ± 1.23

We analyzed the recognition rate and arousal level of dynamic angry faces at different durations (400, 1000, and 1600 ms). There was no significant difference in recognition between the three durations [M ± SD, 400 ms: 0.87 ± 0.034; 1000 ms: 0.84 ± 0.37; 1600 ms: 0.83 ± 0.38, *F*(2, 357) = 0.4, *p* > 0.05]. The ratings for arousal were also not significantly different between the three durations [400 ms: 7.04 ± 1.25; 1000 ms: 6.97 ± 1.23; 1600 ms: 7.00 ± 1.37; *F*(2, 357) = 0.10, *p* > 0.05].

### 2.3. Procedure

Participants were seated in front of a monitor showing experimental stimuli through Eprime-2.0 software (Psychology Software Tools Inc., Pittsburgh, USA). Each participant went through three experimental stages: the initial learning stage, the practice stage, and the last formal test.

In the initial learning phase, participants were randomly presented with a 12 cm × 16 cm pink oval that lasted for one of two different durations—400 ms as the standard short duration and 1600 ms as the standard long duration.

In the second practice phase, participants were presented with a 12 cm × 16 cm pink oval for 400 or 600 ms and asked to judge whether this oval was closer to the short duration or long duration. Feedback was provided as either “correct” or “wrong.” Participants were only able to proceed to the next formal test after their accuracy rate reached 80% or above.

**FIGURE 1:**
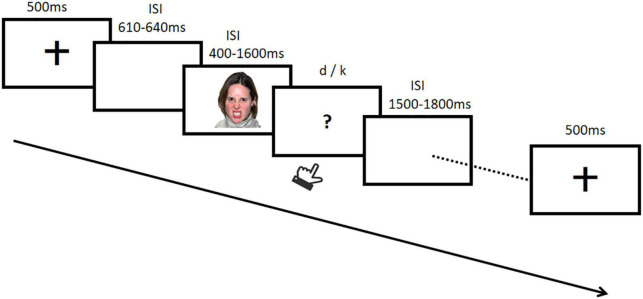
Example of the procedure for a formal test trial.

In the formal test, the oval was replaced with a facial expression of a different emotion type and facial dynamic combinations (static neutral, static anger, and dynamic anger), each last for six different durations (400, 600, 800, 1000, 1200, 1400, and 1600 ms). The procedure of a single trial is depicted in Figure 1. A fixation cross was first shown for 500 ms and then followed by a 610–650 ms inter-stimulus interval, then the facial expression was presented with random duration of 400, 600, 800, 1000, 1200, 1400, or 1600 ms. Participants were asked whether the duration of this expression was closer to the standard short or long duration, and responses were made by pressing “d” (short condition) or “f” (long condition). The button-press assignment was counterbalanced across different participants. No feedback was shown in the formal test. Each participant completed 630 trials in five blocks, each block consisted of 18 facial expression (six pictures for each of the three facial stimuli: static neutral, static anger, and dynamic anger) with seven different durations. The test lasted about 30 min.

### 2.4. EEG recording and analysis

EEG recordings were obtained with NeuroScan system (NeuroScan, Inc., Herndon, VA, USA) from 32 electrodes positioned according to the 10/20 system and referenced to the bilateral mastoid with a bandpass filter of 0.05–30 Hz. An electrooculogram was recorded from electrodes placed below and lateral to the eyes. Curry 7 software was used for offline data processing (Compumedics, Abbotsford, Australia). The time window was chosen from –200 ms before stimulus onset (pre-stimulus 200 ms was used as the baseline) and 1800 ms after the stimulus. Blinks and other eye movement artifacts were removed using independent-component analyses. Six ERP components were separately analyzed based on previous research and our hypotheses. For the N1 and P2 components, the mean amplitudes were separately averaged at three centro-frontal electrodes (Fz, FCz, and Cz) in the 70–140 ms and 150–190 ms time windows, respectively; the same was applied for the EPN at two posterior electrodes (O1 and O2) in the 250–350 ms time window, and for the LPP at two centro-parietal electrodes (CPz and Pz) in the 320–800 ms interval, as well as for the CNV at the centro-frontal electrodes (Fz and FCz) from 250 ms to the end of the stimulus presentation.

### 2.5. Statistical methods

The psychophysical function with the proportion of long responses [*p*(long)] was plotted against the seven different duration in the three different facial dynamic groups. We also computed two temporal parameters to better account for the variations of time perception according to previous studies (Fayolle and Droit-Volet, 2014); namely, the Bisection Point (BP) and the Weber Ratio (WR).

The BP is the subjective point of equality, which is the duration of time the subjects responded with long as often as they did short, *p*(long) = 0.5. The smaller the BP value, the more overvalued the time.

The WR indicates the time sensitivity, and was computed as the result of differential threshold {D[*p*(long) = 0.75]–D[*p*(long) = 0.25]}/2 divided by BP [time duration corresponding to 75% *p*(long)-time duration of 25% *p*(long)]. The lower the WR, the steeper the psychophysical function and the higher the temporal sensitivity.

The *p*(long) was computed as the subjects’ response as “long” divided by the total number of trials. Behaviors were analyzed using the *p*(long) as a dependent variable, and the facial dynamic condition (static neutral, static anger, and dynamic anger) and duration as independent variables.

For the ERP results, a repeated-measures ANOVA was applied to assess differences in the peak and latency of the N1, P2, and N2, and the average amplitude (volatility) of the EPN, LPP, and CNV as the dependent variables, and the facial dynamic condition and electrode position as independent variables.

## 3. Results

### 3.1. Behavioral results

#### 3.1.1. Analysis of the proportion of “long” responses

**FIGURE 2:**
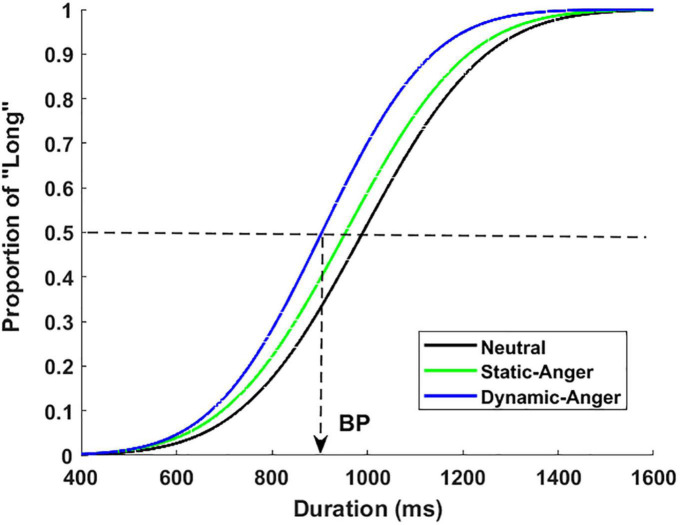
Proportion of long responses plotted against the different durations for dynamic angry, static angry, and neutral faces.

Figure 2 presents the psychophysical functions of the proportion of long responses [*p*(long)] plotted against the seven duration conditions in the three facial dynamic groups. This revealed an important effect of dynamic features on time perception. As shown in this figure, the psychophysical functions shifted more toward the left for dynamic angry faces, compared with the static anger and static neutral faces. An ANOVA was run on the *p*(long) with the duration and dynamic features as within-subjects factors. The results showed a significant main effect of duration, *F*(6, 252) = 380.98, *p* < 0.001, η^2^_*p*_ = 0.90. *Post hoc* analysis suggested that the differences of *p*(long) between different durations were all significant [*M*_400__*ms*_ = 0.03 ± 0.05, *M*_600__*ms*_ = 0.11 ± 0.15, *M*_800__*ms*_ = 0.35 ± 0.22, *M*_1000__*ms*_ = 0.61 ± 0.20, *M*_1200__*ms*_ = 0.81 ± 0.14, *M1*_400__*ms*_ = 0.90 ± 0.10, *M*_1600__*ms*_ = 0.94 ± 010]. The main effect of dynamic features was also significant, *F*(2, 42) = 11.48, *p* < 0.001, η^2^_*p*_ = 0.35, with a *p*(long) that was much higher in the dynamic anger condition than in the static anger and neutral conditions. The *p*(long) in the static anger condition was also significantly higher than that in the static neutral condition (*M*_*DA*_ = 0.63 ± 0.13, *M*_*SA*_ = 0.53 ± 0.18, *M*_*NF*_ = 0.45 ± 0.11). The interaction between duration and facial dynamic features was also significant, *F*(12, 252) = 4.83, *p* < 0.001, η^2^_*p*_ = 0.19. Simple effect analysis revealed that the *p*(long) of the dynamic angry face was significantly longer than that of the static anger and neutral faces in the 800, 100, and 1200 ms conditions (800 ms: *M*_*DA*_ = 0.53 ± 0.27, *M*_*SA*_ = 0.33 ± 0.27, *M*_*NF*_ = 0.19 ± 0.11; 1000 ms: *M*_*DA*_ = 0.82 ± 0.14, *M*_*SA*_ = 0.59 ± 0.29, *M*_*NF*_ = 0.41 ± 0.18; 1200 ms: *M*_*DA*_ = 0.91 ± 0.08, *M*_*SA*_ = 0.81 ± 0.18, *M*_*NF*_ = 0.72 ± 0.17).

#### 3.1.2. Bisection point and weber ratio in the different facial dynamic conditions

An ANOVA revealed a significant main effect of facial dynamic features on the BP, *F*(2, 28) = 9.38, *p* < 0.01, η^2^_*p*_ = 0.40. The BP was lower for the dynamic angry face condition than for the static angry and neutral face conditions, and the BP was significantly lower in the static angry face condition than in the neutral face condition (*M*_*DA*_ = 847.11 ± 135.01, *M*_*SA*_ = 947.30 ± 173.67, *M*_*NF*_ = 1035.06 ± 176.24). The comparison between static angry and neutral faces confirmed a lengthening effect of high-arousing facial expressions (anger in the present experiment). The comparison between dynamic anger and static anger further suggested a lengthening effect when facial expressions were presented dynamically.

In contrast, the ANOVA on the WR did not show any significant results [*F*(2, 34) = 0.33, *p* > 0.05, η^2^_*p*_ = 0.02; Table 2], thus suggesting that time sensitivity was not different between the three facial conditions (dynamic angry, static angry, and static neutral faces).

**TABLE 2:** Bisection point and weber ratio for the three different facial expression conditions.

	Bisection point			Weber ration		
	**Neutral**	**Static anger**	**Dynamic anger**	**Neutral**	**Static anger**	**Dynamic anger**
*M*	1035.06	947.3	847.11	0.10	0.11	0.10
*SD*	176.24	173.67	135.01	0.05	0.04	0.04

#### 3.1.3. Reaction time of different emotion types and facial dynamics

**FIGURE 3:**
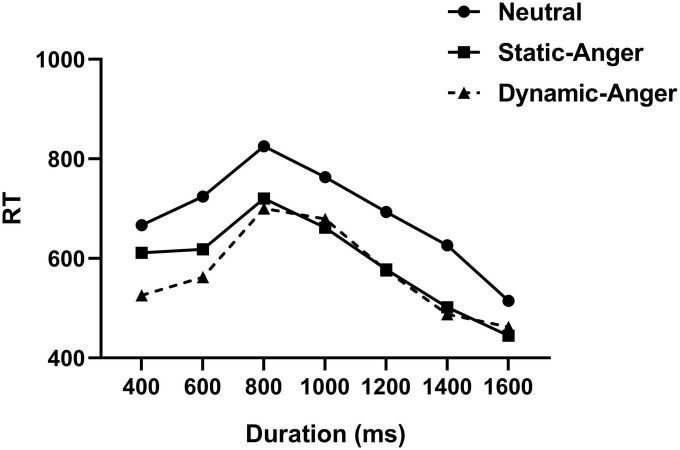
Reaction times in the different facial expression conditions.

An ANOVA was conducted on the reaction time (RT), with duration and facial expression features as within-subject factors. There was a significant main effect of duration on RT, *F*(6, 252) = 25.70, *p* < 0.001, η^2^_*p*_ = 0.38, whereby the RT was significantly longer in the 800 and 1000 ms duration conditions than in the rest conditions (400, 600, 1200, 1400, and 1600 ms). The RT in the 1600 ms condition was significantly longer than in the rest (400, 600, 800, 1000, 1200, and 1400 ms) (*M*_400__*ms*_ = 601.27 ± 155.15, *M*_600__*ms*_ = 634.90 ± 147.12, *M*_800__*ms*_ = 748.70 ± 165.51, *M*_1000__*ms*_ = 701.34 ± 185.49, *M*_1200__*ms*_ = 615.48 ± 167.43, *M1*_400__*ms*_ = 538.51 ± 173.62, *M*_1600__*ms*_ = 474.10 ± 121.51). The main effect of facial dynamic feature on RT was also significant, *F*(1, 42) = 4.11, *p* < 0.05, η^2^_*p*_ = 0.16, whereby the RTs in response to dynamic and static angry faces were significantly shorter than those in the neutral face condition (*M*_*DA*_ = 570.48 ± 148.49, *M*_*SA*_ = 590.75 ± 131.27, *M*_*NF*_ = 687.76 ± 198.47; Figure 3).

### 3.2. ERP results

#### 3.2.1. P2

A 3 (dynamic feature: dynamic anger, static anger, neutral) × 2 (hemisphere: left, right) repeated-measures ANOVA was conducted on the average amplitudes of the P2 component. The main effect of facial dynamic feature was significant, *F*(2, 84) = 13.89, *p* < 0.001, η^2^_*p*_ = 0.25. P2 amplitude evoked by static anger faces was significantly higher than dynamic anger and neutral (*M*_*DA*_ = 3.18 ± 2.53 μV; *M*_*SA*_ = 4.75 ± 2.46 μV; *M*_*NF*_ = 3.17 ± 2.82 μV). The interaction between these two factors was not significant (*p* > 0.05).

#### 3.2.2. N2

**FIGURE 4:**
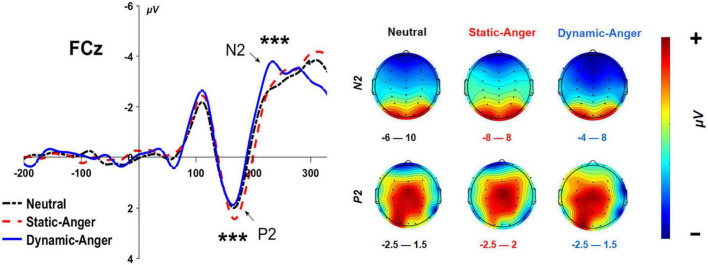
The grand-mean ERP waveforms of the N2 and P2 components at FCz. ***P < 0.001.

A 3 (dynamic feature: dynamic anger, static anger, neutral) × 2 (hemisphere: left, right) repeated-measures ANOVA analysis was also conducted on the average N2 amplitude. There was a significant main effect of a facial dynamic feature on N2 amplitude, *F*(2, 84) = 9.71, *p* < 0.001, η^2^_*p*_ = 0.19, whereby the N2 amplitude evoked by dynamic anger faces was significantly larger than that elicited in the static anger and neutral conditions (*M*_*DA*_ = –2.17 ± 3.08 μV; *M*_*SA*_ = –1.25 ± 3.55 μV; *M*_*NF*_ = –0.84 ± 2.85 μV; Figure 4). There was no main effect of the hemisphere and no interaction between these two factors (*p* > 0.05).

#### 3.2.3. EPN

**FIGURE 5:**
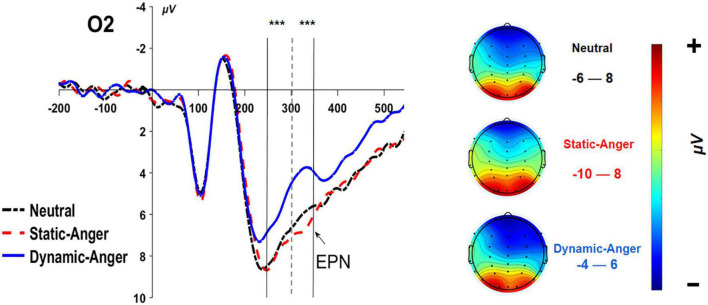
The grand-mean ERP waveforms of the EPN component at O2. ***P < 0.001.

The same ANOVA analysis was conducted on the average EPN amplitude in the 250–300 ms time window. There was a significant main effect of facial dynamic feature on EPN amplitude, *F*(2, 56) = 15.56, *p* < 0.001, η^2^_*p*_ = 0.36, whereby the EPN amplitude evoked by dynamic anger was significantly smaller than that evoked by neutral faces and static anger (*M*_*DA*_ = 5.60 ± 3.30 μV, *M*_*NF*_ = 6.97 ± 4.08 μV, *M*_*SA*_ = 7.40 ± 3.66 μV). The other main effect and interaction effect was not significant (*p* > 0.05). In the 300–350 ms time window, the amplitude induced by dynamic anger, neutral faces, and static anger gradually increased significantly, with dynamic angry faces evoking significantly larger amplitude than neutral faces, neutral faces also induced significantly larger amplitude than static angry faces (*M*_*DA*_ = 3.96 ± 2.42 μV; *M*_*SA*_ = 4.84 ± 3.30 μV; *M*_*NF*_ = 6.07 ± 3.70 μV; Figure 5).

#### 3.2.4. LPP

**FIGURE 6:**
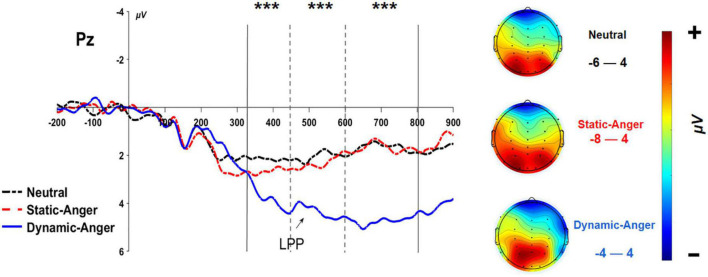
The grand-mean ERP waveforms of the LPP component at Pz. ***P < 0.001.

An ANOVA analysis on the average LPP amplitude in two different time windows (320–450 ms and 450–800 ms) revealed there to be a significant main effect of facial dynamic feature, 320–450 ms: *F*(2, 56) = 24.61, *p* < 0.001, η^2^_*p*_ = 0.47; 450–800 ms: *F*(2, 56) = 47.26, *p* < 0.001, η^2^_*p*_ = 0.63. In the 320–450 ms time window, the amplitude evoked in the dynamic anger condition was significantly higher than that evoked in the static anger and neutral face conditions; the amplitude in the static condition was also significantly larger than that in the neutral face condition (*M*_*DA*_ = 2.82 ± 3.37 μV; *M*_*SA*_ = 1.14 ± 3.68 μV; *M*_*NF*_ = 0.39 ± 2.74 μV). In the 450–800 ms time window, the amplitude induced by dynamic anger was also significantly larger than that elicited in the static anger and neutral face conditions, but there was no significant difference between the static anger and neutral face conditions (*M*_*DA*_ = 4.49 ± 3.27 μV; *M*_*SA*_ = 1.54 ± 3.32 μV; *M*_*NF*_ = 1.27 ± 2.50 μV). The other main effect and interaction effect were not significant (*p* > 0.05; Figure 6).

#### 3.2.5. CNV

**FIGURE 7:**
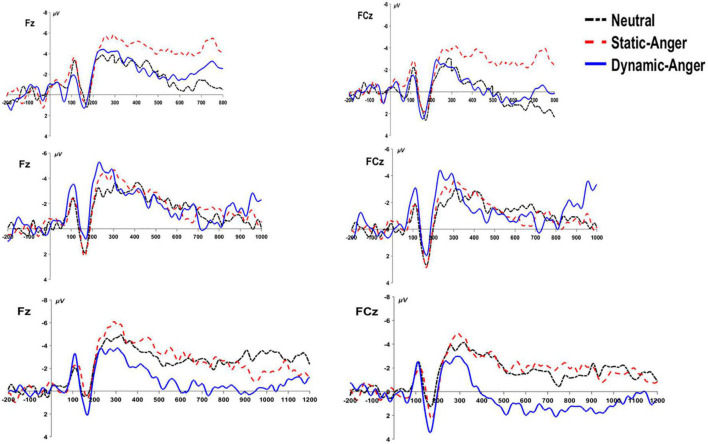
The grand-mean ERP waveforms of the CNV component at Fz and FCz under the three different duration conditions (800, 1000, and 1200 ms).

According to the behavioral results, the *p*(long) was significantly different between the duration conditions of 800, 1000, and 1200 ms. We conducted three ANOVA analyses separately to assess the CNV amplitude under these three duration conditions, using the average amplitude of the CNV from 250 ms to the end of the stimuli presentation. In the 800 ms duration condition, there was a significant main effect of a facial dynamic feature on CNV amplitude, *F*(2, 56) = 18.88, *p* < 0.001, η^2^_*p*_ = 0.24. The CNV amplitude evoked by static anger was significantly larger than that evoked in the dynamic anger and neutral conditions (*M*_DA_ = –0.53 ± 5.08 μV; *M*_SA_ = –4.88 ± 5.06 μV; *M*_NF_ = –1.12 ± 5.46 μV). In the 1000 and 1200 ms duration conditions, there was a significant main effect of a facial dynamic feature on CNV amplitude [1000 ms: *F*(2, 56) = 6.84, *p* < 0.01, η^2^_*p*_ = 0.2; 1200 ms: *F*(2, 56) = 19.09, *p* < 0.001, η^2^_*p*_ = 0.41]. The CNV amplitude in the static anger and neutral face conditions was significantly larger than that in the dynamic anger condition (1000 ms: *M*_DA_ = –0.67 ± 4.36 μV, *M*_SA_ = –2.30 ± 4.21 μV, *M*_NF_ = –1.97 ± 4.21 μV; 1200 ms: *M*_DA_ = 1.33 ± 5.03 μV, *M*_SA_ = –2.65 ± 3.51 μV, *M*_NF_ = –2.83 ± 3.05 μV). No other significant effects were found (*p*s > 0.05; Figure 7).

## 4. Discussion

The central aim of the present study was to investigate the effect of facial dynamic features on time perception using ERP methods. The behavioral results revealed a significant overestimation effect when judging the duration of dynamic angry faces compared with static angry and static neutral faces. Several ERP components were assessed to objectively characterize the states generated by the dynamic features of the facial expression. All five components predicted to be involved (the N2, P2, EPN, LPP, and CNV) showed a significant dynamic effect, but with different amplitude patterns. Taken together, these results confirm our hypothesis that the dynamic feature of facial expression can influence individuals’ time perception both at behavioral and electrophysiological levels.

In terms of behavioral performance, we observed a steady and significant decrease in the *p*(long) judgment from dynamic anger to static anger and neutral faces, which supports previous findings (Fayolle and Droit-Volet, 2014; Li and Yuen, 2015; Xu et al., 2021). According to the scalar timing theory, attention to attractive or arousing stimuli results in the activation of a switch that alters the number of pulses emitted from a pacemaker, which are subsequently collected in an accumulator, and this causes the perceived duration to be prolonged (Gibbon, 1977; Lake, 2016). Second, from the perspective of facial movement, dynamic faces attract more attention due to motion characteristics, which leads to a longer perceived duration than of static faces (Fayolle and Droit-Volet, 2014; Li and Yuen, 2015). Finally, the RT for both dynamic angry and static angry faces was significantly shorter than the RT for neutral faces. This further confirms the arousal effect caused by high-arousing expression (anger) rather than low-arousing expression (neutral) (Gil and Droit-Volet, 2011; Tipples et al., 2015; Uusberg et al., 2018; Benau and Atchley, 2020).

The ERP results suggested that there is an early processing advantage for static angry faces *via* emotional arousal and attentional bias, while dynamic angry faces are mainly processed in a later temporal stage *via* response inhibition and attracting attention. Specially, we found that the P2 amplitude was larger for static angry faces than for dynamic angry and neutral faces, while no significant difference was found between dynamic angry and neutral faces. Previous studies have reported that the P2 might reflect conscious access to sensory information and that it can be modulated by alternative attention. High-arousing and negative angry facial expressions have been proposed to attract more attentional resources, which results in a larger P2 amplitude (Hillyard et al., 1973; Cui and Luo, 2009). Given that the dynamic anger material was morphed from a neutral expression to an increasingly intense angry face with six facial sequences (frames), the earlier time window of 150–190 ms (P2) for dynamic anger was more inclined to a neutral rather than an angry expression. Take the dynamic angry face with duration of 400 ms as example, it consists 6 frames changing gradually from neutral face (the first frame) to the most intense angry face (the sixty frame). While the first half of this dynamic angry face (0 ms–200 ms) was more inclined to be the neutral face. This may account for the reason why no difference in amplitude was found between dynamic anger and neutral face condition.

Similarly, the N2 amplitude was significantly greater for dynamic anger than static and neutral face conditions. Previous studies have found that the frontal located N2 component was more related with odd stimuli and response inhibition (Folstein and Petten, 2008). According to one study employing go/no-go paradigm, the no-go condition evoked a larger N2 amplitude (Folstein and Petten, 2008; Wang et al., 2020). Compared with static faces, dynamic faces have been found to have a greater effect on individuals’ attentional processes. Subjects need to inhibit dynamic information to accomplish a task (Li and Yuen, 2015). In this regard, this larger N2 amplitude in the dynamic anger condition may reflect the inhibition processes (Gan et al., 2009).

The EPN amplitude became significantly larger from the dynamic angry face to neutral and static anger face conditions. As an early posterior negativity, the EPN reflects an increase in the amount of sensory processing resources, which is modulated by brain systems in which visual representations are evaluated in terms of their meaning, such as the amygdala and prefrontal cortex (Pourtois et al., 2012). The EPN amplitude has been found to be significantly different in response to emotional and neutral stimuli (Recio et al., 2011). Furthermore, the emotional effect on EPN is reportedly modulated by the amount of cognitive resources (Perry et al., 2019; Wang et al., 2019). In one previous study, the EPN was not affected by negative facial stimuli and showed a pattern of automatic processing in a rich cognitive resource condition; the emotional effect was only present in the low cognitive resource demanding condition, whereby presentation of a high-arousing picture evoked a larger EPN amplitude (Zhu and Liu, 2014). This result further demonstrated that the emotional effect on EPN was not influenced by attentional control, but an automatic process (Schindler and Kissler, 2016). Furthermore, as explained, in the early time window of 250–300 ms, the dynamic stimuli may be still more inclined to be perceived as neutral face among the six facial sequences (frames) consisting the dynamic stimuli, which may further explain why the dynamic angry face had no effect on time perception.

We also found a significantly larger LPP amplitude in response to dynamic and static anger faces compared with neutral faces (320–450 ms). This result is in line with evidence from previous studies showing that the emotional effect in response to high-arousing stimuli (angry faces) induces a larger LPP amplitude than does low-arousing stimuli (neutral faces) (Schupp et al., 2004; Weinberg and Hajcak, 2010). The LPP component has been identified as a key indicator of the arousal effect on attentional processes, and attention is captured more by negative arousing stimuli than by arousing stimuli of another valence (Ye et al., 2018; Long et al., 2020; Xie et al., 2022). However, in the later 450–800 ms time window, there was no significant difference in LPP amplitude, as a measure of time perception, between the static angry and neutral face conditions, while time perception on dynamic angry faces still evoked a significantly larger amplitude than static angry and neutral faces. This result can be interpreted according to a negative processing advantage that occurs at the early processing stage (320–450 ms), whereby more attentional resources are attracted to angry faces than to neutral faces, which results in the emotion effect (anger vs. neutral faces). In the later processing stage (450–800 ms), the negative processing advantage could have been replaced by the motion characteristics of the dynamic faces. The LPP is a later component that is indicative of a higher analysis level and evaluation of emotion stimuli, which require more cognitive resources. This could be why there was no significant difference in the LPP amplitude caused by time perception of static angry and neutral faces. Another possible explanation is related to the cognitive resources required to perform the present temporal bisection task. Compared with other temporal judgment paradigms, such as the reproduction paradigm, the current study employed the temporal bisection paradigm, which has been shown to be a low cognitively demanding task (Qu et al., 2021). Enough cognitive resources seemed to be allocated to both the temporal judgment task and implicit emotion perception task. In the dynamic anger condition, individuals’ attention was more easily attracted by the motion characteristics of the dynamic angry faces, which may result in more attentional resources available for the implicit emotion perception and evaluation task. More empirical evidence is still needed to verify this possibility.

The CNV amplitude has been interpreted as a marker of temporal accumulation, with longer subjective durations associated with larger amplitudes, and to be related to processes such as arousal level, expectation, and attention (Macar et al., 1999; Macar and Vidal, 2004). In the present study, after analyzing the CNV amplitude under three duration conditions (800, 1000, and 1200 ms) separately, the data consistently suggested that the CNV amplitude induced by static angry faces was larger than that elicited in response to dynamic anger, and did not accumulate with the increase in duration. Given that the arousal level between dynamic and static angry faces was not significantly different based on pre- and post-experiment rating results, the difference in CNV amplitude may reflect different mechanisms underlying the processing of dynamic and static facial stimuli during the temporal bisection task. However, some researchers have also suggested that the complex pattern of CNV was not solely accounted for by emotion arousal and attentional processes, and other studies found no direct relationship between the CNV amplitude and time processing (Kononowicz and Hedderik, 2011; Tamm et al., 2014). Future studies are needed to further investigate the underlying mechanisms and possibilities.

This study has some limitations that should be noted. First, we employed the temporal bisection paradigm to investigate dynamic and emotional effects on time perception. However, previous studies have found that the temporal task used may influence the effect of emotion on time perception (Gil and Droit-Volet, 2011), and different tasks may exert different cognitive demands, which may lead to different results. Further comparisons should be made using different temporal judgment paradigms simultaneously. Second, the psychological meaning of the ERP components found in the present study still requires further evidence. For instance, it is not entirely clear whether the larger N2 amplitude by time perception in response to dynamic angry faces is an indicator of response inhibition or a response to odd stimuli. Finally, the sample size in the present study was relatively small and future research should recruit more subject to further evidence the dynamic effect found in the present study.

## 5. Conclusion

To summarize, using a classical temporal bisection task with different comparison durations and dynamic emotional expressions (dynamic angry, static angry, and static neutral faces), we revealed a significantly different effect of dynamic versus static expressions on time perception. The analysis on the proportion of long responses, BP, and RT results suggested that the duration of dynamic facial expression was overestimated compared with static and neutral expressions. The ERP results indicated that dynamic features evoked different ERP responses. The static angry faces mainly induced larger P2 and EPN components, while the dynamic angry faces evoked larger-amplitude N2 and LPP components. These results indicate that different neural mechanisms may underlying the overestimation effect of time perception between facial expressions with different dynamic features.

## Data availability statement

The raw data supporting the conclusions of this article will be made available by the authors, without undue reservation.

## Ethics statement

The studies involving human participants were reviewed and approved by the Research Ethics Committee of College of Preschool Education, Capital Normal University. The patients/participants provided their written informed consent to participate in this study. Written informed consent was obtained from the individual(s) for the publication of any potentially identifiable images or data included in this article.

## Author contributions

FQ and XS contributed to designing the experiments and analyzing the data. XS, JD, TG, and HW contributed to collecting the data. FQ and CG contributed to writing the manuscript. All authors contributed to the article and approved the submitted version.
